# Dramatic declines in seropositivity as determined with crude extracts of *Plasmodium falciparum* schizonts between 2000 and 2010 in Dielmo and Ndiop, Senegal

**DOI:** 10.1186/1475-2875-13-83

**Published:** 2014-03-06

**Authors:** Fode Diop, Vincent Richard, Babacar Diouf, Cheikh Sokhna, Nafissatou Diagne, Jean-François Trape, Michel Matar Faye, Adama Tall, Gora Diop, Aissatou Toure Balde

**Affiliations:** 1 Institut Pasteur de Dakar, Avenue Pasteur, Dakar, Senegal; 2 Université Cheikh Anta Diop de Dakar, Fann, Dakar, Senegal; 3 Institut de Recherche et de Développement, Dakar, Sénégal

**Keywords:** Malaria, *Plasmodium falciparum*, Seroepidemiology, IgG, Schizont crude extract, Senegal, Dielmo, Ndiop

## Abstract

**Background:**

Programmes of pre-elimination of malaria have been implemented in Senegal since 2010, and the burden of malaria has decreased substantially. These changes in the epidemiology should be monitored with effective tools that allow changes in patterns of transmission to be estimated. In Dielmo and Ndiop, two villages of Senegal with different malaria endemicity, infections have been followed longitudinally for 20 years, during which time there have been several control interventions leading to substantial decreases of transmission. This study aimed to compare malaria antibody responses of the inhabitants of these two villages, between 2000 and 2010, using schizont crude extracts of a local strain of *P. falciparum (Pf Sch*07/03).

**Methods:**

Sera collected from inhabitants of the two villages (141 from Dielmo and 79 from Ndiop in 2000; 143 from Dielmo and 79 from Ndiop in 2010) were used to assess the prevalence of antibodies against crude schizont extracts of *Pf Sch*07/03. Three ages groups were defined: [5-9] yrs, [10-14] yrs and [15-19] yrs. Statistical comparisons were performed. Seroprevalence and the magnitude of antibody responses were compared between age groups, villages and periods.

**Results:**

Overall seroprevalence to *P.fSch07/03* decreased between 2000 and 2010 in both villages: from 94.4% to 44.4% in Dielmo and from 74.4% to 34.6% in Ndiop. The difference between Dielmo and Ndiop was highly significant in 2000 (*p* < *0.001*) but not in 2010 *(p >0.20)*. The decrease in seroprevalence was larger in younger (more than 40%) than older (less than 19%) inhabitants. Longitudinal monitoring of the younger group showed that seroprevalence decreased between 2000 and 2010 in Dielmo from 98.7 to 79.3, but not in Ndiop from 67.6 to 66.7. The magnitude of antibody responses in seropositive individuals was significantly higher in 2000 than 2010 for both villages.

**Conclusions:**

Crude extracts of *P. falciparum* are appropriate tools for evaluating malaria prevalence at different periods, and in both low and high endemic area. Using crude extracts from local strains to assess transmission may allow efficient evaluation of the consequences of control programs on malaria transmission.

## Background

There has been significant progress over the last ten years in the fight against malaria, a major threat to public health. The World Health Organization (WHO) reported that of 102 countries in which transmission continues, 52 recorded a decrease of more than 50% in the number of malaria cases between 2000 and 2010. In parallel, malaria mortality has also decreased by more than 45% globally and, in particular, by 47% in Africa. These results have been obtained by a combination of interventions, including artemisinin-based combination therapy (ACT), extensive coverage of exposed populations with long-lasting insecticide-impregnated bed nets (LLITN), malaria diagnosis by rapid diagnostic tests (RDTs), and intermittent treatment strategies [1]. The changes in malaria epidemiology needs to be monitored through time by control programmes to assess the effects of the strategies implemented and to anticipate the consequences of changing rates of malaria transmission [2]. Additional and appropriate tools are required to evaluate transmission, because parasite prevalence and entomological measures can be insufficiently sensitive in areas of low transmission [3].

Various methods have been used since the 1960s to measure humoral responses to malaria and thereby evaluate malaria seroprevalence and transmission intensity. However, the variability of sources of antigen and subjectivity of some of the detection methods led to these methods falling out of favour [4]. Sensitive enzyme-linked immunosorbent assay (ELISA) remains the most attractive technique for assessment of malaria transmission and changes in prevalence following the establishment of control programmes [5]. Recent studies using characterized antigens have concluded that the evaluation of antibody persistence through cross-sectional surveys could be a complementary or even an alternative approach to investigating markers of malaria [6]. There are however constraints and limitations to serological tests with characterized antigens: immunological responses to a given antigen may be, in part, genetically determined; and some of these antigens have the potential to, or may, display substantial polymorphism [7]. Previous studies have shown that responses to crude extracts of malaria parasites, which are mixtures of numerous antigens, are less sensitive to these constraints [5,8].

The objectives of this study were to assess the value and feasibility of testing immune responses to parasite crude extracts. This approach was used to evaluate changes in malaria transmission over a ten year period in Dielmo and Ndiop, two villages of Senegal where there has been longitudinal follow-up since 1990. Various malaria interventions were implemented between 2000 and 2010, in accordance with the recommendations of the National Malaria Control Programme [9]. These interventions were associated with a large decrease in malaria transmission. The relationship between these changes and local populations antibody responses to crude parasite extracts was investigated.

## Methods

A cross-sectional study was conducted for the years 2000 and 2010 using the sera collected at the end of the wet season, during the Dielmo and Ndiop cohort study described previously [10,11].

### Setting

At the beginning of the cohort studies, (in 1990 for Dielmo and in 1993 for Ndiop), the features of malaria transmission risk differed between the two villages. At that time Dielmo was an area where malaria was holoendemic with substantial activity of the main mosquito vector *Anopheles gambiae sensu lato* complex and, therefore, perennial parasite transmission. By contrast, Ndiop was a mesoendemic area with moderate and seasonal transmission: transmission was 10 times lower than that observed in Dielmo [12,13]. The estimated annual entomological inoculation rate (EIR) in these areas was established periodically during the course of the cohort study. Between 2000 and 2010, the EIR significantly changed in the two villages: 482 infected bites/person/year in 2000 *vs* 88.7 infected bites/person/year in 2010 for Dielmo and 79 infected bites/person/year in 2000 *vs* 4.6 infected bites/person/year in 2010 for Ndiop.

The parasitaemia prevalence in November in the three age groups defined in this study was respectively 48.1, 54.1 and 53.3% in 2000 *versus* 4.9, 8.1 and 8.7% in 2010 in Ndiop. This prevalence was 44.9, 25 and 25% in 2000 *versus* 0, 4.8 and 3.3% in 2010 in Dielmo.

### Population

The study was conducted using sera collected from individuals less than 20 years of age. A total of 442 serum samples from inhabitants of Dielmo and Ndiop collected for the cohort study were included in this study: 220 for 2000 (141 from Dielmo and 79 from Ndiop) and 222 for 2010 (143 from Dielmo and 79 Ndiop). Socio-demographic characteristics for the donors of these sera were extracted from the database established for the cohort study [10,11]. This study was examined and approved by the Senegalese National Health Research Ethics Committee.

### *Plasmodium falciparum* culture and crude extract preparations

The *P. falciparum* strain 07/03 (*Pf* 07/03) was isolated from a patient in Dielmo and adapted to culture in the Immunology Unit of Pasteur Institute in Dakar [14]. Parasites were cultured continuously on O^+^ erythrocytes in RPMI containing 0.5% Albumax in candle jars according to the method described by Trager and Jensen [15]. Crude extracts of schizonts of this strain (Sch07/03) were prepared by the method published by Wahlgren *et al.*[16]. After centrifugation of the culture, one volume of the pellet was lysed in three volumes of sterile distilled water and vortexed for homogenization. The extract was frozen without centrifugation at −80°C in working aliquots.

### ELISA assay

ELISA Maxisorp plates (Nunc, Roskilde, Denmark) were used to optimize the dilution by dose effect. The optimal dilution was 1/300 and this was used for coating. Plates were coated with 100 μl of water-soluble crude extracts of *Pf*07/03 schizonts (Sch07/03). Non-infected red blood cell were diluted in PBS and distributed into some wells on each plate as a control. The coated plates were incubated overnight at 4°C and washed. Then, 200 μl of blocking buffer (2% BSA in PBS with 0.05% Tween) was added to each well and the plates incubated for 1 hour at 37°C. Plasma samples were diluted at 1/200 in a dilution buffer (1% BSA in PBS with 0.05% Tween) and 100 μl of diluted serum was distributed into each well. Negative and positive controls were included on each plate: positive controls were from Dielmo/Ndiop hyperimmune individuals and negative controls were from European individuals. Polyclonal goat anti-human IgG conjugated to peroxidase at a dilution of 1/6,000 in the dilution buffer (1% BSA in PBS-Tween 0.05%) was then added. Bound peroxidase was detected with ortho-toluidine/H_2_O_2_ (100 μl) and the reaction was stopped by addition of 4 N H_2_SO_4_ (50 μl/well). Between each incubation phase, the ELISA plates were washed extensively with PBS-0.05% Tween. The optical density (OD) at 450 nm was read in a BIO-RAD Microplate Reader (iMark). The threshold for positivity was defined as an OD ratio >2 (OD sample/OD naive serum). Inter-assay variations for positive controls did not exceed 20%.

### Statistical analysis

*R* software was used for statistical analysis [17]. Three age groups were defined: [5-9] yrs, [10-14] yrs and [15-19] yrs. The seroprevalence of anti-Sch07/03 antibodies (Abs) was compared between 2000 and 2010 after standardization. Fisher’s exact tests were used to compare categorical variables and antibody levels between the villages and between the years 2000 and 2010. Non parametric tests (Kruskall Wallis, Wilcoxon) and the Spearman test were used to study non-parametric quantitative variables. Differences were considered statistically significant for *p < 0.05.*

## Results

### Demographic characteristics

From the collection established for the cohort study in Dielmo and Ndiop, 224 sera were randomly selected for 2000 and 220 for 2010. Blood samples had been collected during the rainy season in both years. Table 1 summarizes the characteristics of the study population. The mean age was 10.3 years [9.9-10.8] in 2000 and 10.8 years [10.5-11.4] in 2010, with no significant difference between the groups for the two periods (*p = 0.06).* There was also no difference in the sex ratio (57.7% male in 2000 and 51.8% male in 2010).

**Table 1:** Demographic characteristics of the study population

	**2000**		**2010**		***p.Value***
	**N = 222**	**(%)**	**N = 220**	**(%)**	
***Location***					
Dielmo	144	(64.9)	141	(64.1)	0.9
Ndiop	78	(35.1)	79	(35.9)	
***Sex***					
Male	128	(57.7)	114	(51.8)	0.3
Female	94	(42.3)	106	(48.2)	
***Age***					
Mean	10.3		10.9		0.06
95% CI	[9.9-10.8]		[10.5-11.4]		
***Age groups***					
[5-9] yrs	109	(49.1)	87	(39.5)	0.07
[10-14] yrs	88	(39.6)	95	(43.2)	
[15-19] yrs	25	(11.3)	38	(17.3)	

### Seroprevalence of Sch0703 antibodies was significantly lower in 2010 than 2000

**Figure 1:**
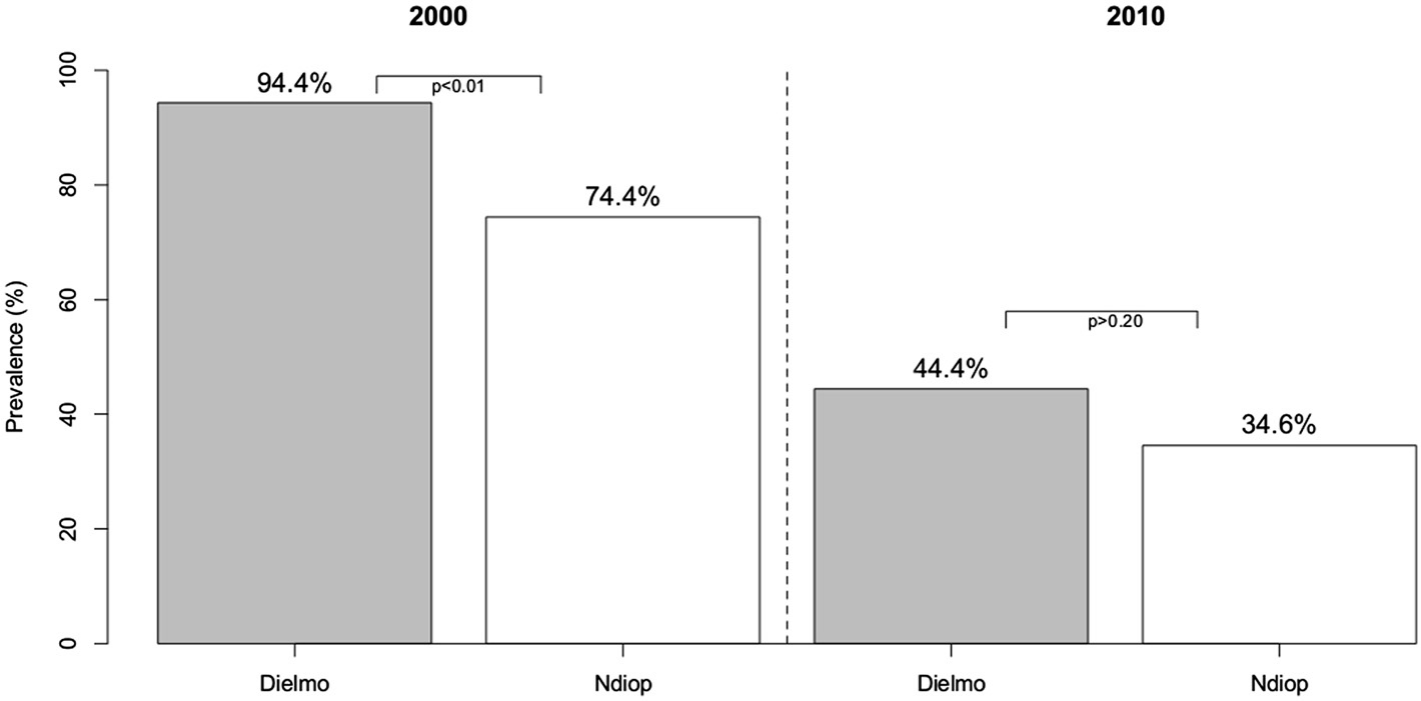
**Seroprevalence of *Sch*** **07/03 Abs in Dielmo and Ndiop after standardization.**

Standardized seroprevalence values were lower in 2000 than 2010, dropping from 94.4% to 44.4% in Dielmo and 74.4% to 34.6% in Ndiop (Figure 1). The difference in the seroprevalence of *Sch07/03* Abs in 2000 between Dielmo and Ndiop was highly significant (*p < 0.001*), but this difference between Dielmo and Ndiop was much smaller, and indeed not significant, in 2010 (*p > 0.20).*

### Changes in seroprevalence of Sch07/03 antibodies, according to age group

**Figure 2:**
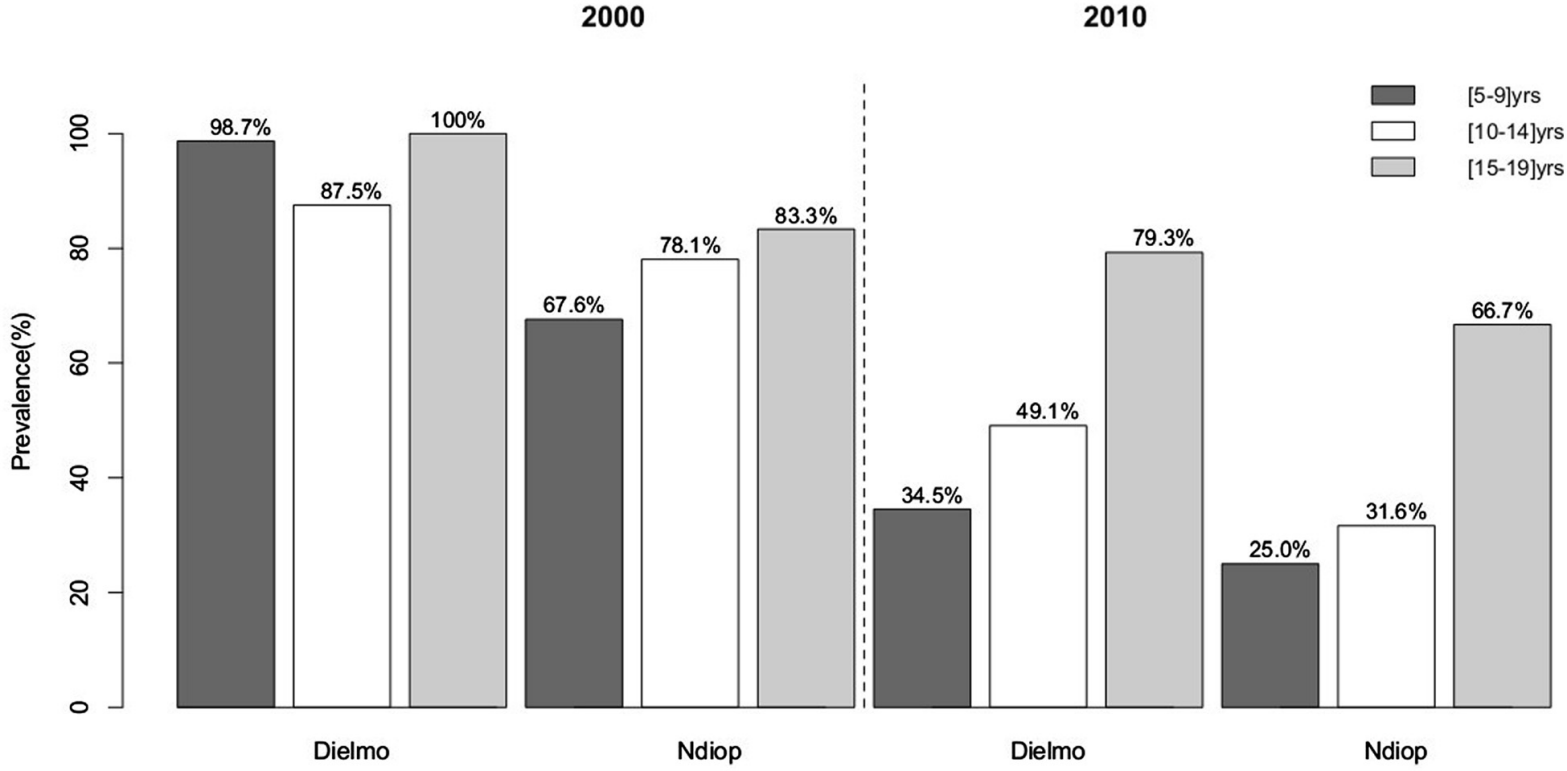
**Antibody responses to crude extracts of *Sch*07/03.** Prevalence percentages by age group after standardization in Dielmo and Ndiop villages during 2000 and 2010.

The prevalence of anti-Sch07/03 antibodies (Abs) was also analysed in various age groups in each village (Figure 2). For all age groups and for both villages, seroprevalence was higher in 2000 than 2010. In 2000, the prevalence of Sch07/03 Abs in Dielmo was high in every age group, and was highest in the oldest group: 98.7% [5-9] yrs, 87.5% [10-14] yrs and 100% [15-19] yrs. In Ndiop, the prevalence of Sch07/03 Abs increased with age group: 67.6% [5-9] yrs, 78.1% [10-14] yrs and 83.3% [15-19] yrs (Figure 2). The prevalence of Sch07/03 Abs was lower in 2010 than 2000 both in Dielmo and Ndiop. In Dielmo the prevalence values in 2010 were 34.6% in the [5-9] yrs age group, 49.1% in the [10-14] yrs age group and 79.3% in the [15-19] yrs age group. In Ndiop the 2010 values were 25.0% in the [5-9] yrs age group, 31.6% in the [10-14] yrs age group and 66.7% in the [15-19] yrs age group.

The 2010 seroprevalence values in the different age groups in Dielmo were similar to those in Ndiop. The seroprevalence had decreased more and more significantly in the younger age group (by more than 40%) than in the older age group (by less than 19%). To study this decrease of seroprevalence, we compared the seroprevalence in the [5-9] yrs age group in 2000 and that ten years later (2010) in the age group ten years older (the [15-19] yrs age group]. In Dielmo, the percentage of positive samples was 98.7% in the [5-9] yrs age group in 2000 to 79.3% in [15-19] yrs age group in 2010. However in Ndiop, the prevalence was unchanged: 67.6% in the [5-9] yrs age group in 2000 and 66.7 in the [15-19] yrs age group in 2010.

### Changes in mean levels of antibodies to Sch07/03, according to age group

**Figure 3:**
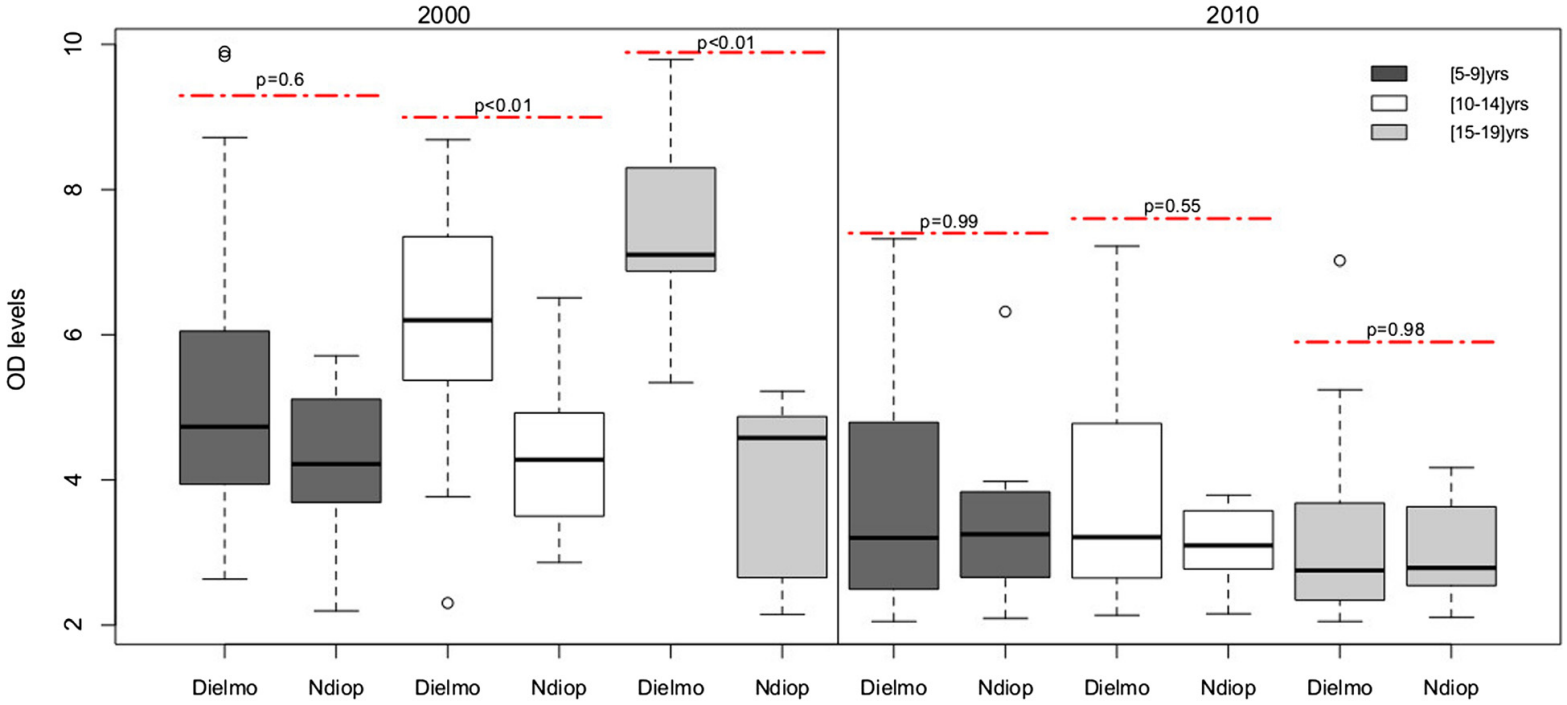
**Antibody responses to crude extracts *Sch*** **07/03 according to age group: comparison of the responses between Dielmo and Ndiop in 2000 and 2010 in positive responders only.**

A shift in the type of malaria transmission might be reflected by the magnitude of the antibody response to *P. falciparum*. To investigate this possible relation, the magnitude of the IgG responses to Sch07/03 was determined in positive responders of the three age groups (Figure 3). The magnitude of IgG responses was high in children less than 10 years old, and increased with age especially in Dielmo in 2000. For all age groups, in 2000, the magnitude of the IgG response was greater in Dielmo (mean OD ratios > 4) than Ndiop. In 2010, the mean levels of antibodies against Sch07/03 were significantly lower than in 2000 (mean OD ratios <3) and no significant difference in responses was found between age groups in the two villages (Figure 3).

**Figure 4:**
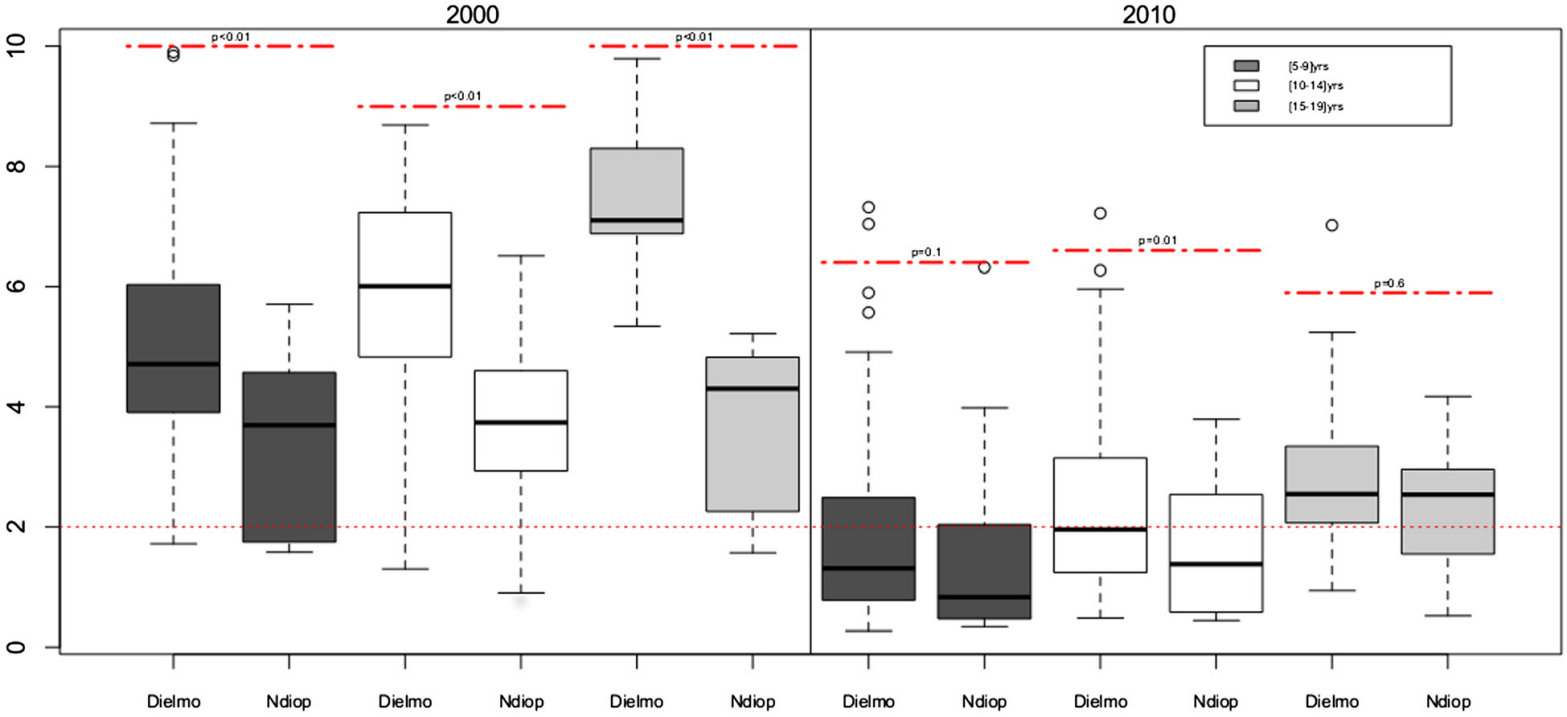
**Antibody responses to crude extracts *Sch*** **07/03 according to age group: comparison of the responses between Dielmo and Ndiop in 2000 and 2010 in all subjects.**

The mean levels of IgG antibodies for all subjects between Dielmo and Ndiop by age group for the two periods were also compared (Figure 4). The differences between Dielmo and Ndiop were statistically significant for 2000: all nominal *p* values were less than 0.001. However for 2010, the mean OD ratios of IgG antibodies did not differ significantly between Dielmo and Ndiop.

The 5–9 years of age group in 2000 was compared with the corresponding the 15–19 years of age group in 2010. The mean IgG antibody OD ratios were significantly higher in the [5-9] group in 2000 than in the corresponding [15-19] group in 2010 in both villages (Figures 3 and 4). This finding contrasts with the finding that the prevalence in Ndiop did not differ between these groups (Figure 2).

## Discussion

The objectives of this study were to investigate the prevalence of anti-malarial antibodies and the levels of antibodies to crude extract of schizonts of a local *P. falciparum* strain. The ideal test for detecting malarial antibodies in epidemiological studies would be specific, give reproducible results and could be used at large scale. Therefore, the ELISA method was used as a technique suitable for high throughput and reproducible testing. Another advantage of this technique is its low cost, facilitating generalized and standardized use in the areas where it is needed.

Many seroepidemiological analyses have used a single recombinant antigen to evaluate serological responses. This strategy does not favour sensitivity and may lead to substantial underestimation of the immune response to *P. falciparum* infection. Antigenic polymorphism and the diversity of individual responsiveness can have large effects on the serological responses observed, and thus on the results obtained with recombinant antigens [18,19]. By contrast, crude extracts containing numerous antigens may allow greater sensitivity and thus detection of low-level residual transmission. ELISA based on crude extracts is, therefore, more informative and a better tool for following endemicity [5,20].

In this study a schizonts crude extract of *Pf*Sch07/03 was used in the ELISA as a tool to measure and compare immune responses and seroprevalence. Antibody responses against this crude extract of *Pf*Sch07/03 of subjects recruited in the villages of Dielmo and Ndiop for antibodies were analysed.

This study was conducted in the villages of Dielmo and Ndiop. These villages were selected because the epidemiological contexts were different. Dielmo was holoendemic with a perennial and high transmission, with 200 to 300 infected bites/person/year [13] whereas Ndiop was a mesoendemic area with moderate and seasonal transmission (during the rainy season between September and December), with 20 to 30 infected bites/person/year (10 fold less than in Dielmo). However, by 2010, the EIR had decreased to 79 infected bites/person/year in Dielmo and to 4.6 infected bites/person/year in Ndiop.

Seropositivity to *PfSch07/03* crude extracts was tested among the inhabitants of Dielmo and Ndiop for the years 2000 (220 subjects) and 2010 (222 subjects). The prevalence of anti-*P. falciparum* Abs differed between Dielmo and Ndiop in 2000. The seroprevalence to *Pf*Sch07/03 was high for all age groups studied in Dielmo, and highest in young adults (around 100%) in 2000. This high seroprevalence to *Pf*Sch07/03 is consistent with the high levels of exposure in this region of perennial transmission and holoendemicity [21]. The seroprevalence in Ndiop increased with age in 2000 and was at 83.6% for 15–19 yrs older group. This reflects the seasonal transmission and the delay in acquiring an immune response as described by Perraut *et al.*[22]. These findings are in agreement with seminal epidemiological reports [23-25]. Studies conducted before the introduction of intensive malaria control programmes reported a higher prevalence and protective role of antibodies in the endemic Dielmo and Ndiop areas [21] and West African countries more generally [26]. The seroprevalence had declined substantially after 10 years in both Dielmo (44.4%) and Ndiop (34.6%), such that they were similar in the two villages in 2010; this is presumably a consequence of the anti-malarial strategies implemented since 2008 in Senegal [27]. There was no significant difference in seroprevalence between males and females in Dielmo or Ndiop, in either year (*p* > 0.05). These findings are in agreement with previous epidemiological studies [28].

Differences in the increases and decreases of antibody prevalence between age groups were also considered. Seroprevalence decreased further and more significantly in the youngest group (by more than 40%) than the oldest group (less than 19%). There is probably a delay before acquiring an immune response in younger individuals, whereas adults display a ‘memory effect’ and their immune response can persist as a consequence of a history of contact with the parasite [29,30]. This could be due to the change of endemicity in Dielmo where transmission has declined following implementation of control programmes [27,31]. Similar findings have also been reported in other western African countries in 2013 [32]. The analyses of the anti- *Pf*Sch07/03 IgG response in the youngest age group in 2000 (corresponding to the 15–19 year-old group in 2010) revealed another interesting feature of malaria transmission and seroprevalence: the percentage of positive subjects in Dielmo was significantly lower 10 years later, suggesting that adults had, by 2010 lost the immune response to malaria antigens that they had had when they were ten years younger. By contrast, in Ndiop the seroprevalence was unchanged after 10 years in the same group. This observation might reflect the low intensity of transmission, especially among adults, in Ndiop.

The mean levels of anti- *Pf*Sch07/03 Abs decreased substantially between 2000 and 2010 in both Dielmo and Ndiop. These declines were such that the magnitude of antibody responses did not differ between the two villages in 2010. Although seroprevalence appears to have remained constant over 10 years in Ndiop, the mean levels of Abs responses were highly significantly different between the younger group in 2000 and the corresponding older group in 2010. The decrease of the antibodies levels was comparable in Dielmo and Ndiop suggesting that a similar mechanism was operating to reduce malaria prevalence in the two villages. Trape *et al.* reported a longitudinal follow up of inhabitants of Dielmo, showing that the incidence density decreased after LLITN distribution [31]. Recent reports in Gambia demonstrate that these policies substantially reduce malaria morbidity, mortality and prevalence [26]. The present study argues that sero-epidemiology could be a valuable additional monitoring tool particularly in the context of pre-elimination of malaria. The standardization by different laboratories of this method based on using crude extracts of *P. falciparum* in assessments of the overall anti-blood-stage immune responses would make this approach even more useful for monitoring exposure to malaria.

## Competing interests

The authors declare that they have no competing interests.

## Authors’ contributions

AT conceived and coordinated this study. FD and AT conceived the manuscript. FD VR, GD and AT designed the methodology, conducted the analyses and wrote the manuscript and BD, MF, CS, JT, ND and AdT contributed to developing the concepts presented in this paper and provided valuable insights during the revision and editing stages. All authors read and approved the final manuscript.
